# Contralateral Routing of Signal Devices Reduce Objective and Subjective Listening Effort in Unilateral Cochlear Implant Recipients

**DOI:** 10.1097/ONO.0000000000000078

**Published:** 2025-10-13

**Authors:** Sara Neumann, Sabrina Calise, Bob Dwyer, Smita Agrawal

**Affiliations:** 1 Hearts for Hearing, Oklahoma City, Oklahoma; 2 Advanced Bionics, Clinical Research, Valencia, California.

**Keywords:** Asymmetric, Cochlear implant, CROS, Hearing loss, Listening effort

## Abstract

**Objective:**

This study investigated the impact of a contralateral routing of signal (CROS) device on subjective and objective listening effort and speech recognition in unilateral cochlear implant (CI) recipients.

**Study Design:**

A single-group, prospective, repeated measures design in 2 technology conditions (with CROS and without CROS), 3 talker locations (0°, 90°, and 270°), and 3 signal-to-noise ratio conditions (quiet, easy, and hard).

**Setting:**

Nonprofit audiology and speech language therapy (listening and spoken language) program

**Participants:**

Unilateral adult CI recipients with limited functional hearing in their poorer performing ear or bilateral CI listeners willing to participate with their better hearing or preferred implanted ear for the duration of the testing (N = 15).

**Intervention:**

Participants were fitted with a CROS device on their poorer hearing ear.

**Main Outcome Measures:**

Objective listening effort measured via verbal response time, subjective listening effort and motivation measured via questionnaires, and speech recognition.

**Results:**

The CROS device reduced objective and subjective listening effort and improved speech recognition, particularly when speech was presented to the non-CI ear.

**Conclusions:**

The CROS device can reduce subjective and objective listening effort and improve speech recognition and motivation in certain situations.

While cochlear implants (CIs) have revolutionized the treatment of moderate sloping to profound hearing loss (HL) (1), unilateral cochlear implantation poses significant challenges to auditory recognition, sound localization, and speech understanding, especially in environments where the signal of interest originates from the nonimplanted ear or when the less favorable signal-to-noise ratio (SNR) is presented to the CI side (2,3). If the nonimplanted ear has limited aidable hearing, does not respond well to amplification, or cannot be implanted, traditional hearing aids might not be effective. In such cases, a contralateral routing of signal (CROS) microphone offers a nonsurgical solution by routing sound from the poorer to the better hearing side, overcoming the negative impacts of the head shadow and improving auditory perception and speech recognition outcomes (4–10), perceived quality of life (11), and could also have implications for cognitive processes, such as listening effort.

Individuals with HL often report that listening is effortful, highlighting its clinical significance. It is distinct from but related to speech understanding (12). Listening effort can be defined as the allocation of attentional and cognitive resources toward detecting, decoding, processing, and understanding spoken words or other auditory signals (12,13). It has been shown that individuals with HL must increase mental effort compared with those without HL when attempting to detect, process, and respond to auditory stimuli (14,15). In the lab, listening in high levels of noise (ie, poor SNR) increases listening effort in adults (16–18) and children (18–21) and contributes to listening-related fatigue (22), which is important considering the nonfinite nature of cognitive resources (23) and that cognitive demand (influenced by HL and task demands) and motivation are key contributors to listening effort (12).

In the real world, school-aged children with unilateral HL are already at a higher risk for poorer speech, language, and cognitive outcomes compared with their typical hearing peers. Children with limited usable hearing unilaterally (LUHU; (24–26)) or single-sided deafness face even greater challenges, including even poorer speech recognition outcomes and a greater need for academic support than children with less severe unilateral HLs (27). Additionally, these children exert significantly more effort in listening, especially in noisy environments, already exacerbating challenging academic experiences (28). In adults, the consequence of increased listening effort is equally troublesome and includes increased mental fatigue, reduced quality of life, and reduced work performance (29–32). It is reasonable to expect that the effect will be similar, if not more pronounced, in individuals with a unilateral CI and LUHU in the contralateral ear.

A frequently employed behavioral approach for assessing listening effort and cognitive load is via a dual-task paradigm. When gauging listening effort, the main task is typically speech recognition. Meanwhile, a secondary task often encompasses a memory or nonauditory activity, such as a visual or motor response task. The deterioration in performance (eg, accuracy or response speed) observed in the secondary task during multitasking is interpreted as an indication of cognitive resources being reallocated from the secondary task to the primary task.

Single-task assessments are employed less frequently for the assessment of listening effort and cognitive load. In a single-task paradigm, 2 aspects of the same task are measured. In the context of a speech recognition task, this means recording the correctness of the responses and assessing the speed at which the responses were given. Single-task paradigms have a distinct advantage over dual-task paradigms in that they avoid issues related to controlling task priorities and allocation of resources inherent in dual-task designs. Recent work has shown that a single-task paradigm, such as a verbal response time (VRT) measure, which quantifies the time delay between presentations of a speech stimulus and the spoken response, can assess listening effort and cognitive load, providing a clearer understanding of the cognitive demands placed on adults and children with HL (19–21,33–35).

The benefit of a CROS device for speech understanding in specific listening situations is well documented (4–9,36–38), but the impact of CROS use on subjective and objective listening effort is less known. Oosthuizen et al (39) employed behavioral and subjective measures of listening effort to investigate the benefit of CROS hearing aids in school-age children 7–12 years old, with LUHU. With the CROS device, children demonstrated less listening effort, as measured via VRT, when the signal was presented toward the CROS side (ie, the indirect condition). Specifically, 89% of participants demonstrated faster (shorter) VRTs with the CROS system, indicating a reduction in cognitive load during listening tasks. Additionally, subjective ratings revealed that the CROS system contributed positively to the children’s perceived ease of listening and motivation to engage in auditory tasks. These findings suggest that CROS technology can effectively reduce listening effort in children with LUHU, highlighting their potential utility in educational settings.

This study seeks to add to the literature by investigating the effects of CROS on speech understanding and listening effort in adult unilateral CI listeners whose contralateral, unimplanted ear does not benefit from amplification (as determined subjectively by the individual and/or audiological assessment). The study objectives are as follows: 1) to measure change in speech understanding in noise with the addition of a CROS device; 2) to assess objective listening effort (via VRT) among participants using a CROS device; and 3) to assess perceived listening effort, ease, and motivation among participants using a CROS device. Since much of the previous work in this area has shown that improvements in speech intelligibility (eg, SNR) show reduced response times, it was hypothesized that 1) the benefit (subjective and objective) of the CROS device will depend on the location of the target talker (CROS × talker location interaction). For example, speech presented toward the non-CI ear will be perceived more accurately with CROS, but not when target speech is presented from the front or toward the better ear; 2) the benefit of the CROS device (eg, speech recognition, subjective and objective listening effort) will vary depending on the presence and level of noise (SNR × CROS interaction); 3) the CROS device will be detrimental when the target speech is presented to the CI side in the presence of noise (SNR × talker location × CROS interaction).

## MATERIALS AND METHODS

This study employed a within-subjects, mixed-methods approach, combining speech recognition tests, VRT measurements, and qualitative assessments (eg, questionnaires). Assessments were conducted under different device configurations in diffuse noise: unilateral CI-only and CI + CROS (default balance setting). The order of each condition was counterbalanced to avoid potential order effects.

### Participants

The study was conducted in accordance with the ethical standards outlined in the *Declaration of Helsinki*. The study was reviewed and approved by an independent institutional review board before participant recruitment. All participants were served by the same nonprofit audiology clinic and enrolled in the study after obtaining written informed consent.

This study recruited CI listeners who, if unilateral, 1) had limited aidable hearing defined as thresholds greater than 70 dB HL (0.125–8 kHz) and for whichever reason(s) had decided not to undergo bilateral cochlear implantation; b) individuals with some aidable hearing, but who do not derive significant benefits from amplification in the nonimplanted ear, or c) bilateral listeners willing to participate with their better hearing or preferred implanted ear only for the duration of the testing. To be considered for study inclusion, participants needed to be recipients of Advanced Bionics CII/90K/Ultra cochlear implants (AB; Valencia, CA), have at least 6 months of CI use experience, be current users of a Marvel CI sound processor, have no experience with the CROS P-13 device, and be fluent in spoken English (as materials are in English). Exclusion criteria included cognitive impairment, as indicated by chart review or NIH Toolbox cognitive screener (40), a sentence recognition score in quiet with the better or preferred ear at 65 dBA of less than 30%, and an inability to participate in speech testing or to follow and complete questionnaires.

### Test Environment, Stimuli, and Equipment

Speech testing was completed in a large room (24’10” × 20’4” × 9’) with an ambient noise level of ~32 dB(A) and reverberation time of 0.4 seconds (RT60). The target stimuli were AzBio sentences (41) presented at 65 dB(A) from 0°, 90°, and 270° (calibrated at the location of the participant’s head). The interferer was diffuse, uncorrelated classroom noise (42) presented continuously throughout the testing from the room’s four corners.

### Procedure

All participants were programmed by experienced, licensed CI programming audiologists. They listened with their everyday program with omnidirectional mic and scene classification systems disabled. Before testing, an aided speech reception threshold (SRT) was obtained for each participant, and all SRTs were confirmed to be no worse than 25 dB HL.

### Outcome Measures

#### Speech Recognition

AzBio sentence recognition was completed with and without the CROS system in quiet and at 2 SNRs. First, an individualized SNR to achieve ~50% of each participant’s intelligibility in quiet was determined by manually adjusting the noise level in the unilateral listening configuration (without the CROS device), with the target speech presented from the front speaker. This SNR is referred to as the “hard” SNR going forward in this article. A second test SNR, referred to as the “easy” SNR, was also used, where the noise level was reduced by 4 dB relative to a given participant’s SNR_hard_. This noise level was chosen for several reasons: 1) prior research has indicated that speech intelligibility levels that are too low may not be sensitive to changes in listening effort (43,44); 2) to align with prior literature, which has employed fixed SNR paradigms using 4 dB intervals (19,45–47); and 3) to limit ceiling effects.

Each test condition was assessed using 1 list (20 sentences per list). Participants were instructed to repeat everything they heard and encouraged to guess if unsure, keeping their heads still and facing the front loudspeaker at 0° throughout testing. Speech recognition scores are reported in a percentage correct of the total words in a given list.

#### Behavioral Listening Effort: Verbal Response Time

Changes in VRT provided a measure of listening effort. Listening effort based on VRT was quantified as the time elapsed (in ms) from the end of the target stimulus to the onset of the verbal response when performing the primary task (AzBio sentence recognition). Responses were recorded from 4 remote microphones (1 placed at each speaker [n = 3] and 1 around the participant’s neck). The microphones were connected to a PC running Audacity (version 3.4.1) to record the stimuli and the participant’s responses, which were used to determine the VRT from the recorded audio and visualization of the acoustic waveforms. Speech fillers and other nonspeech sounds preceding a sentence (eg, “ahhh,” “ummmm,” stutters, and mouth noises) were not regarded as the onset for the VRT. In these instances, the self-corrected second utterance was considered the onset (19,39). In situations where the participant could not respond to something uncontrolled (eg., coughing and sneezing), the duration of these uncontrollable actions did not count toward the VRT. If a participant’s response was stopped or corrected, the VRT onset was considered to be the start of the corrected second utterance (19). VRT from both correct and incorrect trials were included as it would result in better representation of the varying levels of listening effort individuals might experience in everyday life (39,48). The study team routinely convened to review trials that VRT coders felt required additional review.

#### Self-reported Questionnaires

Participants provided perceived listening effort ratings immediately following each sentence list. Similar to previous work, participants were instructed to rate listening effort, ignoring overall task difficulty, fatigue, or concentration effort after each listening condition on a 9-point Likert scale with responses: “1—no effort,” “3—low effort,” “5—moderate effort,” “7—high effort,” and “9—very high effort” (49,50). Given that a listener’s motivation also affects listening effort (12), after each condition, participants were asked if the CROS system made it easier for them to listen (ease of listening) and if the CROS system helped them to keep trying. (motivation) by a simple yes or no response.

### Statistical Analysis

A repeated measures analysis of variance (ANOVA) was used to analyze the effects of the independent variables on the dependent variables. The independent variables included listening configuration (ie, CI-alone and CI + CROS), talker location (CI ear, front, and non-CI ear), and SNR (quiet, easy, and hard). Three dependent variables were assessed: speech recognition score, VRT, and self-reported effort. All statistical analyses were conducted using R version 4.4.0 (51) using the “ez” package version 4.4-0 (52) to carry out the ANOVAs and the “emmeans” package version 1.10.2 (53) to examine interactions. The Wilcoxon signed-rank test from the “stats” package version 4.4.0 (51) was used to compare the perceived listening effort with and without the device for each SNR and talker location combination. An alpha level of 0.05 was used for all statistical tests, and adjusted *P* values (*P*.adj) are reported where corrections were applied. Unless otherwise noted, Tukey method was used to adjust *P* values for multiple comparisons. All plots were created with the “ggplot2” package version 3.5.1 (54).

## RESULTS

The participants (8 female, 7 male) aged 17–78 years (*M* = 52, SD = 20.72) had no experience with a CROS device. Three participants were unilaterally implanted, and 12 bilaterally implanted, participating with only their better performing, or preferred, ear. Of the unilateral listeners, none used a contralateral hearing aid in their everyday listening configuration. All participants had at least 6 months of experience with their implant in the ear tested (*M* = 18 years, SD = 5.235). Additional group demographics are displayed in Table 1.

**TABLE 1.** Participant demographics

	Mean ± SD/n (%)
Age, y	52 ± 20.72 (range: 17–77)
Sex at birth	Male: 7 (47%)/female: 8 (53%)
Ethnicity	White: 15 (100%)
Age at implantation, y	33 ± 21.29 (range: 1–62)
Duration of implant exp., y	18 ± 5.24 (range: 4–24)
Bilateral/unilateral CI	Bilateral: 12 (80%)/unilateral: 3 (20%)
Implanted devices	
HiRes Ultra 3D	1 (6.7%)
CII	4 (26.7%)
HiRes 90K Adv.	2 (13.3%)
HiRes 90K	8 (53.3%)

CI indicates cochlear implant.

### Speech Recognition

**FIG. 1.**
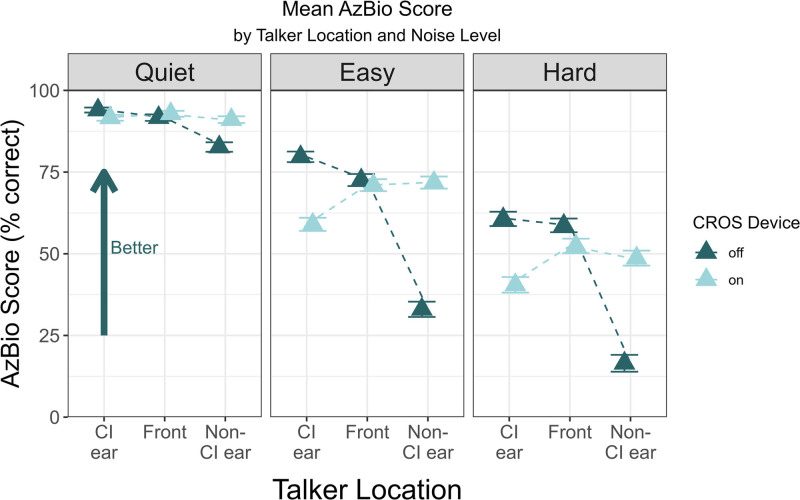
Results of AzBio sentence recognition (% correct) when speech was presented towards the CI ear, front, and non-CI ear in quiet, easy, and hard noise. The mean scores with the CI + CROS and CI-only listening conditions are indicated by differently colored triangles. The error bars represent ±1 SE. CI indicates cochlear implant; CROS, contralateral routing of signal. SE indicates standard error.

Speech recognition results with and without the CROS device for each talker location and noise condition are shown in Figure 1. For bilateral participants, the term “non-CI ear” refers to speech presented toward the side where CI was not used for testing; for unilateral recipients, this is the unimplanted ear. The mean (SD) test SNR for the easy noise condition was 5.9 dB (1.87 dB), with a range of 4–9 dB. For the hard noise condition, the mean SNR was 1.9 dB (1.87 dB), with a range of 0–5 dB.

A repeated measures ANOVA was conducted to examine the effects of CROS, talker location, and SNR on speech recognition scores. The results revealed significant main effects of CROS (*F*(1, 14) = 24.49, *P* < 0.001, partial eta squared (*η*_*p*_*²*) = 0.0386), talker location (*F*(2, 28) = 125.45, *P* < 0.001, *η*_*p*_*²* = 0.4406), and SNR (*F*(2, 28) = 638.59, *P* < 0.001, *η*_*p*_*²* = 0.8396). There were also significant interactions between CROS and talker location (*F*(2, 28) = 205.58, *P* < 0.001, *η*_*p*_*²* = 0.5191), CROS and SNR (*F*(2, 28) = 5.68, *P*.adj = 0.019, *η*_*p*_*²* = 0.0240), talker location and SNR (*F*(4, 56) = 17.61, *P* < 0.001, *η*_*p*_*²* = 0.1412), and a large 3-way interaction effect between CROS, talker location, and SNR (*F*(4, 56) = 34.33, *P* < 0.001, *η*_*p*_*²* = 0.2267). Mauchly test of sphericity indicated that the assumption of sphericity was violated for CROS × SNR interaction; therefore, Greenhouse–Geisser corrections were applied.

A simple effects analysis using estimated marginal means (EMMs) with pairwise comparisons was conducted to explore the strong 3-way interaction. Results are displayed in Tables 2 and 3. In the CI-alone condition, speech recognition was worse when speech was presented to the non-CI ear compared with the front or CI ear. In the CI + CROS configuration, speech recognition improved at the non-CI ear, and differences across locations were resolved. However, when speech was presented to the CI ear in noise, speech recognition worsened, and some differences across talker location were observed. All pairwise comparisons of SNR at each combination of listening configuration and talker location (not shown in tables) were highly significant (ie, *P* < 0.001).

**TABLE 2.** Speech recognition testing results: pairwise comparisons of the EMMs for each talker location at each listening configuration and SNR combination

Comparison	Configuration	SNR	EMM difference	95% confidence interval (lower to upper)	*P*
CI ear vs front	CI-alone	Quiet	2.07	(−4.94 to 9.09)	0.8315
**CI ear vs non-CI ear**	**CI-alone**	**Quiet**	**13.20**	**(6.18 to 20.22**)	**0.0008**
**Front vs non-CI ear**	**CI-alone**	**Quiet**	**11.13**	**(4.11 to 18.15**)	**0.006**
CI ear vs front	CI + CROS	Quiet	−1.48	(−8.5 to 5.54)	0.9101
CI ear vs non-CI ear	CI + CROS	Quiet	0.31	(−6.70 to 7.33)	0.9958
Front vs non-CI ear	CI + CROS	Quiet	1.80	(−5.22 to 8.81)	0.8709
CI ear vs front	CI-alone	Easy	7.93	(0.91 to 14.95)	0.0709
**CI ear vs non-CI ear**	**CI-alone**	**Easy**	**57.27**	**(50.25 to 64.28**)	**<0.0001**
**Front vs non-CI ear**	**CI-alone**	**Easy**	**49.34**	**(42.32 to 56.36**)	**<0.0001**
**CI ear vs front**	**CI + CROS**	**Easy**	**−13.76**	**(−20.78 to −6.75**)	**0.0005**
**CI ear vs non-CI ear**	**CI + CROS**	**Easy**	**−12.30**	**(−19.32 to −5.29**)	**0.002**
Front vs non-CI ear	CI + CROS	Easy	1.46	(−5.56 to 8.48)	0.9125
CI ear vs front	CI-alone	Hard	4.29	(−2.73 to 11.31)	0.4556
**CI ear vs non-CI ear**	**CI-alone**	**Hard**	**49.15**	**(42.13 to 56.16**)	**<0.0001**
**Front vs non-CI ear**	**CI-alone**	**Hard**	**44.85**	**(37.84 to 51.87**)	**<0.0001**
**CI ear vs front**	**CI + CROS**	**Hard**	**−16.27**	**(−23.28 to −9.25**)	**<0.0001**
**CI ear vs non-CI ear**	**CI + CROS**	**Hard**	**−10.20**	**(−17.22 to −3.19**)	**0.0132**
Front vs non-CI ear	CI + CROS	Hard	6.06	(−0.95 to 13.08)	0.2101

Significant differences are indicated in bold.

CI indicates cochlear implant; CROS, contralateral routing of signal; EMM, estimated marginal mean; SNR, signal-to-noise ratio.

**TABLE 3.** Speech recognition testing results: pairwise comparisons of the EMMs for each listening configuration at each combination of talker location and SNR

Comparison	Azimuth	SNR	EMM difference	95% confidence interval (lower to upper)	*P*
CI-alone vs CI + CROS	CI ear	Quiet	2.29	(−4.73 to 9.31)	0.5241
CI-alone vs CI + CROS	Front	Quiet	−1.27	(−8.29 to 5.75)	0.7233
**CI-alone vs CI + CROS**	**Non-CI ear**	**Quiet**	**−10.60**	**(−17.62 to −3.58**)	**0.0034**
**CI-alone vs CI + CROS**	**CI ear**	**Easy**	**22.72**	**(15.70 to 29.74**)	**<0.0001**
CI-alone vs CI + CROS	Front	Easy	1.02	(−6.0 to 8.04)	0.7756
**CI-alone vs CI + CROS**	**Non-CI ear**	**Easy**	**−46.86**	**(−53.88 to −39.84**)	**<0.0001**
**CI-alone vs CI + CROS**	**CI ear**	**Hard**	**26.14**	**(19.12 to 33.16**)	**<0.0001**
CI-alone vs CI + CROS	Front	Hard	5.58	(−1.44 to 12.60)	0.1205
**CI-alone vs CI + CROS**	**Non-CI ear**	**Hard**	**−33.21**	**(−40.23 to −26.19**)	**<0.0001**

Significant differences are indicated in bold.

CI indicates cochlear implant; CROS, contralateral routing of signal; EMM, estimated marginal mean; SNR, signal-to-noise ratio.

### Verbal Response Time

**FIG. 2.**
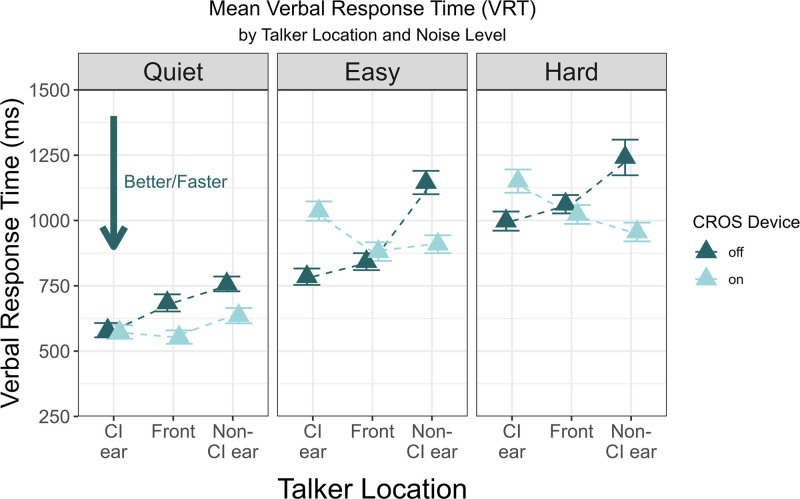
Results of verbal response time (VRT) (in ms) when speech was presented towards the CI ear, front, and non-CI ear in quiet, easy, and hard noise. The mean VRT with the CI + CROS and CI-only listening conditions are indicated by differently colored triangles. The error bars represent ±1 SE. CI indicates cochlear implant; CROS, contralateral routing of signal.

VRT results with and without the CROS device for each talker location and SNR are shown in Figure 2. Observations with response time >5 seconds were omitted from the analyses (35). A repeated measures ANOVA was conducted to examine the effects of CROS, talker location, and SNR on VRT. The results revealed significant main effects of talker location (*F*(2, 28) = 5.29, *P* = 0.011, *η*_*p*_*²* = 0.0257) and SNR (*F*(2, 28) = 72.36, *P* < 0.001, *η*_*p*_*²* = 0.3181). There were also significant interactions between CROS and talker location (*F*(2,28) = 17.63, *P* < 0.001, partial *η*_*p*_*²* = 0.0615), and a 3-way interaction of CROS, talker location, and SNR (*F*(4,56) = 2.81, *P* = 0.03, *η*_*p*_*²* = 0.0152).

A simple effects analysis using EMMs with pairwise comparisons was conducted to explore the moderate effect of the 3-way interaction on VRT. The results are displayed in Tables 4–6. In the easy noise condition with the CI-alone, VRT was significantly slower when the signal was presented to the non-CI hearing ear, compared with the front and CI ear. These differences were resolved in the CI + CROS listening configuration (Table 4). In noise conditions, VRT was faster in the CI + CROS configuration than in the CI-alone condition when the signal was presented to the non-CI ear, in both easy and hard noise conditions. However, VRT was slower when speech was presented toward the CI ear in the easy noise condition (Table 5). When comparing across noise conditions (Table 6), results indicate that comparisons between quiet and both noise conditions consistently show lengthened VRT (compared to in quiet), highlighting the detrimental effects of increased noise levels on auditory performance regardless of configuration (eg, CI-alone and CI+CROS) and talker location (CI ear, front, and non-CI ear).

**TABLE 4.** VRT results: pairwise comparisons of the EMMs for each talker location at each listening configuration and SNR combination

Comparison	Configuration	SNR	EMM difference	95% confidence interval (lower to upper)	*P*
CI ear vs front	CI-alone	Quiet	−104.40	(−306.28 to 97.48)	0.5701
CI ear vs non-CI ear	CI-alone	Quiet	−181.20	(−383.08 to 20.68)	0.1865
Front vs non-CI ear	CI-alone	Quiet	−76.90	(−278.78 to 124.98)	0.7369
CI ear vs front	CI + CROS	Quiet	19.60	(−182.28 to 221.48)	0.9803
CI ear vs non-CI ear	CI + CROS	Quiet	−62.50	(−264.38 to 139.38)	0.8173
Front vs non-CI ear	CI + CROS	Quiet	−82.10	(−283.98 to 119.78)	0.7062
CI ear vs front	CI-alone	Easy	−65.20	(−267.08 to 136.68)	0.8029
**CI ear vs non-CI ear**	**CI-alone**	**Easy**	**−358.40**	**(−560.28 to 156.52**)	**0.0017**
**Front vs non-CI ear**	**CI-alone**	**Easy**	**−293.20**	**(−495.08 to 91.32**)	**0.0134**
CI ear vs front	CI + CROS	Easy	156.40	(−45.48 to 358.28)	0.2849
CI ear vs non-CI ear	CI + CROS	Easy	127.00	(−74.88 to 328.88)	0.436
Front vs non-CI ear	CI + CROS	Easy	−29.40	(−231.28 to 172.48)	0.9561
CI ear vs front	CI-alone	Hard	−69.10	(−270.98 to 132.78)	0.7812
CI ear vs non-CI ear	CI-alone	Hard	−238.10	(−439.98 to −36.22)	0.0566
Front vs non-CI ear	CI-alone	Hard	−169.00	(−370.88 to 32.88)	0.2317
CI ear vs front	CI + CROS	Hard	128.10	(−73.78 to 329.98)	0.4299
CI ear vs non-CI ear	CI + CROS	Hard	184.20	(−17.68 to 386.08)	0.1764
Front vs non-CI ear	CI + CROS	Hard	56.10	(−145.78 to 257.98)	0.8495

Significant differences are indicated in bold.

CI indicates cochlear implant; CROS, contralateral routing of signal; EMM, estimated marginal mean; SNR, signal-to-noise ratio; VRT, verbal response time.

**TABLE 5.** VRT results: pairwise comparisons of the EMMs for each listening configuration at each combination of talker location and SNR

Comparison	Azimuth	SNR	EMM difference	95% confidence interval (lower to upper)	*P*
CI-alone vs CI + CROS	CI ear	Quiet	6.95	(−194.93 to 208.83)	0.9463
CI-alone vs CI + CROS	Front	Quiet	130.93	(−70.95 to 332.81)	0.2055
CI-alone vs CI + CROS	Non-CI ear	Quiet	125.73	(−76.15 to 327.61)	0.2241
**CI-alone vs CI + CROS**	**CI ear**	**Easy**	**−258.94**	**(−460.82 to −57.06**)	**0.0127**
CI-alone vs CI + CROS	Front	Easy	−37.34	(−239.22 to 164.54)	0.7177
**CI-alone vs CI + CROS**	**Non-CI ear**	**Easy**	**226.44**	**(24.56 to 428.32**)	**0.0291**
CI-alone vs CI + CROS	CI ear	Hard	−162.51	(−364.39 to 39.37)	0.1164
CI-alone vs CI + CROS	Front	Hard	34.67	(−167.21 to 236.55)	0.7371
**CI-alone vs CI + CROS**	**Non-CI ear**	**Hard**	**259.77**	**(57.89 to 461.65**)	**0.0124**

Significant differences are indicated in bold.

CI indicates cochlear implant; CROS, contralateral routing of signal; EMM, estimated marginal mean; SNR, signal-to-noise ratio; VRT, verbal response time.

**TABLE 6.** VRT results: pairwise comparisons of the EMMs for each SNR at each listening configuration and talker location combination

Comparison	Configuration	Azimuth	EMM difference	95% confidence interval (lower to upper)	*P*
Quiet vs SNR (easy)	CI-alone	CI ear	−202.8	(−404.68 to −0.92)	0.1227
**Quiet vs SNR (hard**)	**CI-alone**	**CI ear**	**−417.9**	**(−619.78 to −216.02**)	**0.0002**
SNR (easy) vs SNR (hard)	CI-alone	CI ear	−215.0	(−416.88 to −13.12)	0.0952
**Quiet vs SNR (easy**)	**CI + CROS**	**CI ear**	**−468.7**	**(−670.58 to −266.82**)	**<0.0001**
**Quiet vs SNR (hard**)	**CI + CROS**	**CI ear**	**−587.3**	**(−789.18 to −385.42**)	**<0.0001**
SNR (easy) vs SNR (hard)	CI + CROS	CI ear	−118.6	(−320.48 to 83.28)	0.4847
Quiet vs SNR (easy)	CI-alone	Front	−163.6	(−365.48 to 38.28)	0.2535
**Quiet vs SNR (hard**)	**CI-alone**	**Front**	**−382.6**	**(−584.48 to −180.72**)	**0.0007**
SNR (easy) vs SNR (hard)	CI-alone	Front	−219.0	(−420.88 to −17.12)	0.0874
**Quiet vs SNR (easy**)	**CI + CROS**	**Front**	**−331.9**	**(−533.78 to −130.02**)	**0.0042**
**Quiet vs SNR (hard**)	**CI + CROS**	**Front**	**−478.8**	**(−680.68 to −276.92**)	**<0.0001**
SNR (easy) vs SNR (hard)	CI + CROS	Front	−146.9	(−348.78 to 54.98)	0.3299
**Quiet vs SNR (easy**)	**CI-alone**	**Non-CI ear**	**−380.0**	**(−581.88 to −178.12**)	**0.0008**
**Quiet vs SNR (hard**)	**CI-alone**	**Non-CI ear**	**−474.7**	**(−676.58 to − 272.82**)	**<0.0001**
SNR (easy) vs SNR (hard)	CI-alone	Non-CI ear	−94.7	(−296.58 to 107.18)	0.6296
**Quiet vs SNR (easy**)	**CI + CROS**	**Non-CI ear**	**−279.3**	**(−481.18 to −77.42**)	**0.0198**
**Quiet vs SNR (hard**)	**CI + CROS**	**Non-CI ear**	**−340.6**	**(−542.48 to −138.72**)	**0.0031**
SNR (easy) vs SNR (hard)	CI + CROS	Non-CI ear	−61.3	(−263.18 to 140.58)	0.8231

Significant differences are indicated in bold.

CI indicates cochlear implant; CROS, contralateral routing of signal; EMM, estimated marginal mean; SNR, signal-to-noise ratio; VRT, verbal response time.

### Subjective Listening Effort

**FIG. 3.**
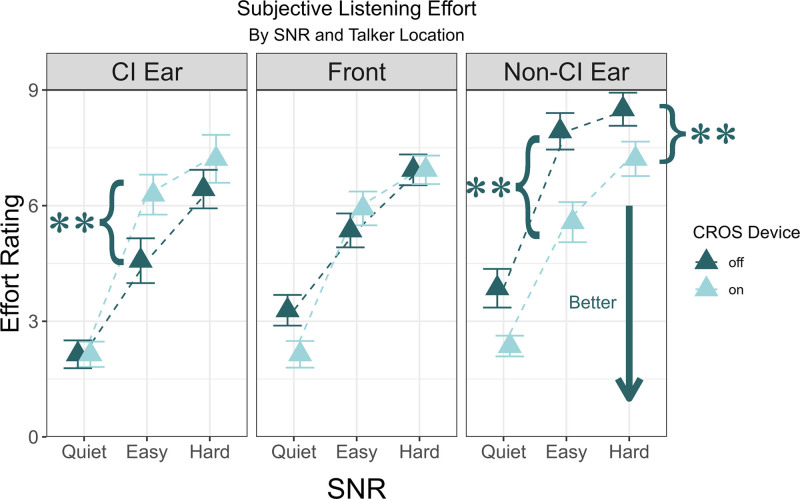
Subjective listening effort when speech was presented in quiet, easy, and hard noise by talker location. The mean ratings with the CI + CROS and CI-only listening conditions are indicated by differently colored triangles. The error bars represent ±1 SE. CI indicates cochlear implant; CROS, contralateral routing of signal.

Results from the self-reported listening effort questionnaire are reported in Figure 3. For the conditions in quiet, no significant difference in listening effort was observed between CI-alone and CI + CROS listening for any combination of SNR and talker location (*P* > 0.05). However, there were significant differences in perceived listening effort between listening configurations for some combinations of SNR and talker location in noise. Specifically, with CI + CROS, when the signal was presented toward the non-CI ear, listening effort was rated significantly lower in easy noise (*W*-value = 0, *P* = 0.003) and hard noise (*W*-value = 0, *P* = 0.005). When the signal was presented to the better ear at the easy noise level, listening effort was rated significantly higher in the CI + CROS configuration, *W*-value = 64.5, *P* = 0.005.

### Subjective Ease and Motivation

**FIG. 4.**
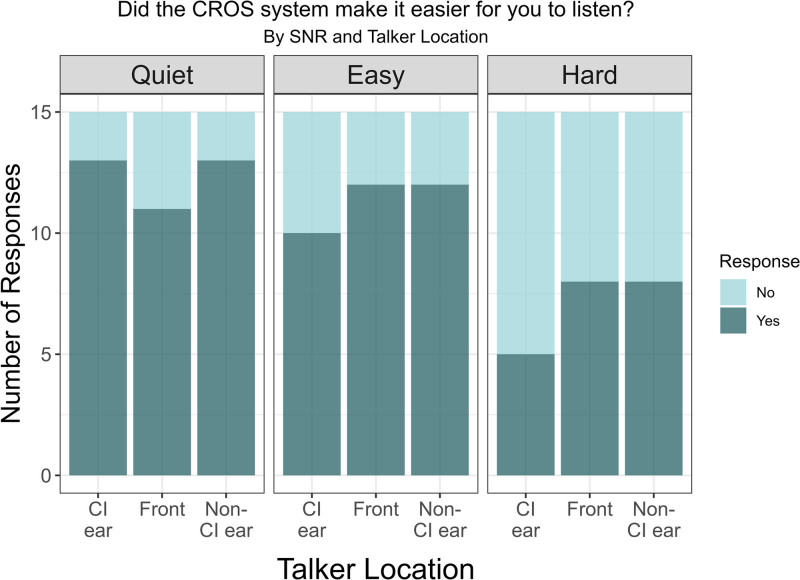
Ease of listening. Responses to the question “Did the CROS system make it easier for you to listen?” categorized by talker location (CI ear, front, and non-CI ear) and listening conditions (quiet, easy, and hard). The bars represent the number of responses, with darker shades indicating “Yes” and lighter shades indicating “No.” CI indicates cochlear implant; CROS, contralateral routing of signal.

**FIG. 5.**
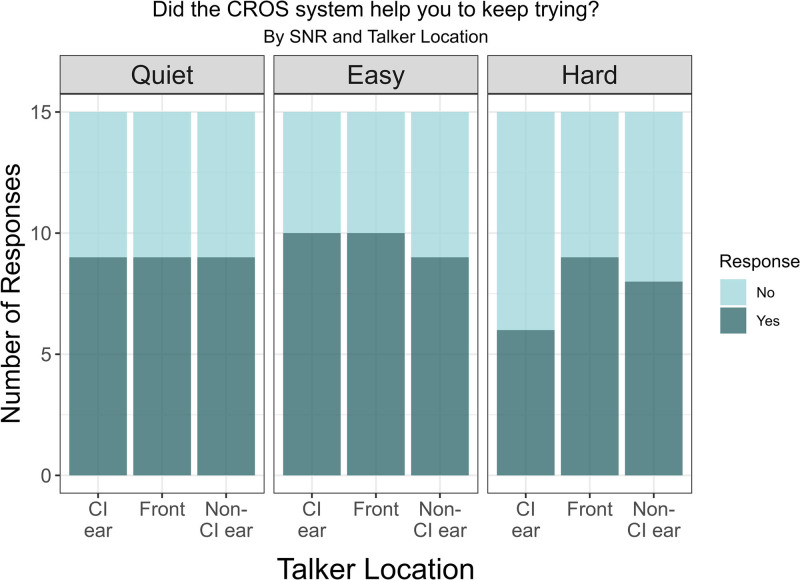
Motivation to participate. Responses to the question “Did the CROS system help you to keep trying?” categorized by talker location (CI ear, front, and non-CI ear) and listening conditions (quiet, easy, and hard). The bars represent the number of responses, with darker shades indicating “Yes” and lighter shades indicating “No.” CI indicates cochlear implant; CROS, contralateral routing of signal.

Figures 4 and 5 report results from the self-reported ease and motivation questions, respectively. Nearly all participants reported that the CROS device made listening easier in quiet and easy noise conditions, with some impact of talker location on the number of participants responding “yes.” When listening in the more difficult SNR, responses to this question were more divided, and the talker location factor was more pronounced, with more “yes” responses when the talker was located at the front or CROS side.

When asked if the CROS system helped motivate the individual to keep trying, over half of the participants reported that it did, particularly in the easier listening conditions with little impact of talker location. However, at the more difficult noise level, the responses were more mixed and impacted by talker location. For example, when the signal was at the CI ear in the most challenging noise level, only 40% of participants (6 out of 15) reported that the CROS motivated them to keep trying to listen to what was being said; however, when the talker was positioned at the front, 60% of participants (9 out of 15) indicated that the CROS system helped them keep trying to listen. In comparison, 53% of participants (8 out of 15) reported a similar motivation when the signal was directed toward the non-CI ear.

## DISCUSSION

Here we demonstrate, for the first time, that a CROS device can reduce objective and perceived listening effort and improve speech recognition in unilaterally implanted adults, particularly when speech is presented to the non-CI ear. While adding a hearing aid (55) or a second implant may reduce listening effort (56), some individuals with a unilateral CI cannot benefit from contralateral ear amplification or implantation. These findings align with our hypotheses that the objective and subjective benefits will be influenced by the target talker’s location, as indicated by the statistically significant CROS × talker location interaction. Notably, we found a detriment to listening effort and speech recognition when the target speech was presented toward the CI side in the presence of noise (SNR × talker location × CROS interaction). We also hypothesized and observed that the benefit of the CROS device will vary depending on the presence and level of the background noise.

Additionally, we investigated subjective ease of listening and motivation. These questionnaire data illustrate how the self-perceived effectiveness of the CROS system varies across different environments and talker locations at highly ecologically valid SNRs—field studies have reported older adults are most often in environments that range from +2 to +14 dB SNR (57). When asked if the CROS system made it easier for them to listen, many participants reported “yes” in the quiet and easy noise conditions, suggesting that they felt the CROS system effectively made their listening experience easier in more favorable listening conditions. When asked if the CROS device helped them to keep trying during the task, the responses were more mixed and more impacted by talker location. Still, these results are encouraging, with many participants reporting that the technology encouraged continued effort across various environments and talker locations at ecologically valid SNRs.

The observed reductions in listening effort and improvements in speech recognition suggest that the CROS device effectively improves listening effort and speech recognition in realistic listening environments. Our findings, and those of others, support the notion that the spatial location of the target talker plays a critical role in the efficacy of the CROS device. Additionally, the variability in benefit based on noise presence and level highlights the importance of knowing when and how to use hearing solutions, such as a CROS device.

The findings in this study align with previous research on VRT in children and adults with HL. Specifically, when in more challenging acoustic listening situations such as those characterized by poorer SNR and decreased intelligibility, VRT is lengthened, suggestive of greater listening effort in these situations (19,21,35,39). Additionally, we observed the benefit of CROS technology on subjective assessments of effort, ease, and motivation. In line with these results, Oosthuizen et al (39) reported subjective ratings that indicated a CROS device positively influenced children’s perceived ease of listening and motivation to engage in auditory tasks. Together, findings suggest that CROS technology can effectively reduce listening effort in individuals with LUHU in the nonimplanted ear.

These results provide a new understanding of the CROS device’s benefits in the domain of listening effort in adults with CIs. These results are of value to audiologists, hearing care professionals, and the recipients they serve, specifically in how they educate their patients about the expected benefit of CROS technology. By knowing the specific conditions under which these devices are most beneficial, practitioners can educate patients to maximize the expected benefits of the technology.

### Limitations and Future Directions

The study limitations identified here should be considered as opportunities for future investigations. As only one CI manufacturer has a CROS device for unilateral CI recipients, the study could only focus on individuals implanted with Advanced Bionics devices, which may limit the generalizability of its findings. The potential benefits of the use of the CROS mute button feature on the CROS P-13 device were not explored in the study. The mute function could improve outcomes by allowing users to manage background noise more effectively when effort and speech recognition are adversely affected by the talker location in noisy conditions. Future studies should investigate how this feature impacts listening effort and speech recognition when intelligibility is lower due to decreased SNR. Finally, the present study examined the effects of acute use of the CROS device. Long-term effects and adaptations to the device were not assessed, which may influence the overall benefits experienced by recipients over time.

## CONCLUSIONS

The CROS device can significantly improve speech recognition and reduce subjective and objective listening effort for unilateral CI listeners. More specifically:

- CROS reduces objective and subjective listening effort and improves speech recognition, particularly when speech is presented to the non-CI ear.
- CROS benefit depends on both the location of the speech signal and the noise level.
- The CROS system can make the user’s listening experience easier and keep them motivated to listen in more challenging listening conditions.

With the absence of an automatic algorithm to modulate the CROS activity based on the location of the dominant speech signal, the benefits of the current CROS system are situationally dependent. Therefore, appropriate patient education and training are necessary to obtain optimal benefit from CROS devices.

## ACKNOWLEDGMENTS

We thank Advanced Bionics for providing the sound processors and CROS devices to support our research and the participants for their time and efforts.

## FUNDING SOURCES

This work was supported by a research grant from Advanced Bionics.

## CONFLICT OF INTEREST STATEMENT

Advanced Bionics employed B.D. and S.A. during this clinical investigation. The remaining authors declare no conflicts of interest.

## DATA AVAILABILITY STATEMENT

The data sets analyzed during the current study are available from the corresponding author on reasonable request.
